# Tasteless Medicines

**Published:** 1860-06

**Authors:** J. W. Thompson

**Affiliations:** Philadelphia


*Tasteless Medicines.* By J. W. Thompson, M. D., Philadelphia.

"Conservative Surgery" and "Rational Medicine" have been deservedly attracting a large share of attention within the last few years; and as a most important branch of the latter, I beg leave to call attention, in a few words, to the subject of "tasteless medicines," a subject which the desire for handsome and reliable preparations, has kept, until recently, entirely too much in the back-ground. Occasionally an effort has been made, and something accomplshed by some one who has been put to his wit's end by the weak stomach, or obstreperous character of a wilful patient, who has literally refused to take his nauseous prescriptions, choosing, instead, to run all the hazards of disease.

But only to a very limited extent have been these improvements, and the sick are still required to take such doses as a well person would loathe to utter detestation.

I am fully persuaded that such should not be the case, now that Chemistry and Pharmacy are being so wonderfully developed; and think that the taste of nearly every article in the Materia Medica may be either nullified or masked, without interfereing at all with its essential properties.

When this cannot be done as a fluid preparation, it can generally be as a jelly, or a solid; the latter in pilular or granular form, coated with sugar, and avoiding that, to many, sickening "Glycerrhiz. Pulv.," in which not a few continue to put up pills, because it answers the purpose, and because their fathers did so.

A great help in this needed reform, is the advancement which has been made in the preparation of the concentrated extracts, and in the separation of the vegetable alkaloids.

It would have been a fruitless task to have attempted, with many of the crude drugs as originally administered, but now is within reach of every hand; and let us see to it, one and all, that this step which is to be the next great improvement in medicine, is taken right early.

In fact we have no right, as humanitarians, to ask a patient to swallow nauseous doses, when we can minister to them in their afflictions, without offending the most delicate palate.

A few I know, who are grounded in the good old ways of antiquity, will say that it is all "fudge"; and that when persons get sick, they ought to be thankful for anything that will do them good. But by such prejudices we ought not, and will soon find that we *must not*, be guided. And those who hold them, will, ere long, be classed in the same category with the surgeons who maintained that pain under their operations was a good thing; and therefore opposed the use of anæsthetics.

In the prosecution of this work (which I am happy to find is already occupying the attention of some of our Pharmaceutists,)

I would throw in a caution as to the *size* of pills. It is easier, generally, to give two, or even half a dozen small ones, than to administer a *bolus;* which, if not a relic of barbarism, is, at all events, rather barbarous treatment.

As to the alkaloids, I am looking forward with confident anticipation to the time when we shall have in this form the active principles of nearly all our vegetable remedies, and hail every one brought forward, as the harbinger of brighter days in store.—And I do so because I see in these a greater certainty of nature and action, a more manageable form, and, paradoxical as it may appear, a less dangerous article; because here we can know what, and what strength, we are giving, which either with the crude drug, or the old extracts, would often resolve itself into a very difficult problem, unless ascertained by experimental administration of each individual specimen. And this surely is not a very satisfactory practice.

Then as we have in this form the principles of a goodly number of our best medicines, let us use them; and at the same time urge on our researches until we can command the whole in the same manner; and once fairly tried, they will not willingly thereafter be done without, by any member of the profession.—*Medical and Surgical Reporter.*

